# Suicide risk characteristics of vocational college students: A latent profile analysis

**DOI:** 10.1371/journal.pone.0333303

**Published:** 2025-10-31

**Authors:** Xiaochun Luo

**Affiliations:** Department of Student Affairs, Sichuan College of Architectural Technology, Chengdu, China; UCL: University College London, UNITED KINGDOM OF GREAT BRITAIN AND NORTHERN IRELAND

## Abstract

**Background:**

Guided by the stress-diathesis model, this study employed latent profile analysis to investigate heterogeneity in suicide risk profiles and inform targeted intervention strategies among college vocational students.

**Methods:**

Data were collected from 1,620 vocational college students identified as high-risk for suicide. Validated instruments—including the Adolescent Life Events Scale (ASLEC), Symptom Checklist-90 (SCL-90), and Social Support Rating Scale (SSRS)—were used to assess stress factors (negative life events), symptom factors (depression, anxiety, psychosomatic symptoms), diathesis traits (neuroticism, adverse childhood experiences), and protective factors (social support). Latent profile analysis (LPA) was applied to identify distinct risk subgroups.

**Results:**

LPA revealed three distinct risk subgroups: a High-risk group (17.4%), characterized by severe psychological symptoms, elevated suicide preparation, and impaired social functioning; a Moderate-risk group (46.5%), defined by neuroticism, persistent despair, and intermediate symptom severity; and a Low-risk group (36.1%), distinguished by robust social support and minimal psychopathological manifestations. Regression analyses indicated that negative life events, depressive symptoms, and neuroticism significantly predicted suicide risk, while social support served as a protective factor.

**Conclusions:**

These findings validate the stress-diathesis framework and advance suicide prevention research by operationalizing heterogeneous risk profiles through LPA. The tripartite classification system offers actionable insights for tiered campus mental health interventions, suggesting crisis management for high-risk individuals, resilience-building for moderate-risk groups, and preventive support for low-risk populations.

## 1. Introduction

Suicide poses a significant global public health challenge, with approximately 740,000 individuals losing their lives to suicide annually. Notably, the suicide rate among young people aged 15–29 is particularly high, ranking it as the third leading cause of death within this demographic [1]. Higher vocational college students fall precisely within this age group. Nevertheless, they are confronted with a multitude of challenges, including academic stress, complex interpersonal relationships, and the daunting task of career planning. These multifaceted pressures have led to a growing prominence of mental health issues among college students. Evidence suggests that this population exhibits a relatively elevated risk of suicide. [2] Suicide risk refers to the possibility of an individual showing suicide behavior in a specific situation, including suicide ideation, attempted suicide, and suicide death. The early identification, assessment, management, and follow-up of individuals at risk of suicide are of vital importance for effective suicide crisis prevention. In the context of college mental health work, how to screen out college students at risk of suicide as early and as scientifically rigorous as possible stands as the paramount priority. The rate of suicide risk detection is contingent upon the assessment tools utilized. Suicide risk assessment refers to the systematic process of evaluating an individual’s likelihood of engaging in suicidal behavior. Its purpose is to identify suicide risk factors, assess the severity of the risk, and develop appropriate intervention strategies. Craig Bullington et al. (2023) noted a 12.5% suicide risk assessment rate [3]. Simon et al. (2013) reported a 12.1% suicide risk detection rate when using Item 9 of the Patient Health Questionnaire-9 (PHQ-9) to assess suicidal ideation [4] Suicidal ideation refers to the thoughts of serving as the agent of one’s own death. Su Binwang et al. (2024) found a higher rate of 17.52% in their assessment of Chinese college students [5].

Based on the enrollment plans of various Chinese universities in 2024, the number of freshmen admitted is approximately between 4,000 and 10,000. Utilizing a assessment rate of 12.5% for 10,000 students, it is estimated that 1,250 individuals will be deemed at risk. This number indicates a large group. The challenge lies in precisely identifying and effectively intervening with these individuals, which demands continuous exploration. Specifically, how to scientifically classify and manage the individuals identified through assessment is the key problem this research aims to address. A survey of 11,288 college students demonstrates that the incidence of suicidal ideation within one year is 17.1%, and the incidence of suicidal behavior within half a year is 3.8% [6]. This implies that in universities, the scale of the suicide-risk group is substantial, and precise identification and classification management are imperatively needed.

The stress-diathesis model of suicide serves as the theoretical foundation for developing a comprehensive assessment approach in this study. The stress-diathesis model of suicide (Stress-Diathesis Model) is a multidisciplinary theoretical framework that integrates physiological, psychological, and social factors to elucidate the mechanisms of suicidal behavior. Initially proposed by Mann et al. (1999), this model posits that suicidal behavior is the result of the dynamic interplay between intrinsic susceptibility factors and extrinsic stressors [7]. Stress factors encompass a wide range of stressors that individuals encounter throughout their life course. These stressors can elicit psychological stress responses in individuals and thereby augment the risk of suicidal behavior. Empirical evidence has consistently demonstrated significant associations between various stress factors and suicidal ideation. For instance, academic pressure, strict parenting styles, and interpersonal relationship difficulties have all been shown to be significantly correlated with suicidal ideation [8–10]. Diatheses refer to inherent susceptibility traits that may be influenced by genetic, biological, psychological, or early experiential factors. Common diathetic factors include impulsivity, low self-esteem, negative cognitive patterns, and neuroticism [7]. Individuals with elevated levels of neuroticism are more prone to suicidal ideation when confronted with negative life events [11]. Protective factors refer to buffering mechanisms that mitigate the interaction between stressors and diatheses. These factors include social support, psychological resilience, and positive coping styles [7]. Research has demonstrated that peer support and teacher social support can reduce the likelihood of suicidal ideation among adolescents [12].

A fundamental goal of suicide prevention is to effectively monitor and provide early warnings for individual psychological crises, enabling timely interventions to prevent severe outcomes. Previous studies often treated college students as a homogeneous group when exploring suicide risk factors, with most focusing on the impact of one or a limited number of risk factors. Notably, suicide crises in real-world settings typically result from the interplay of multiple factors rather than a single determinant. This limitation has led to issues such as high false-positive rates when using single indicators to screen at-risk populations in college student psychological assessments. To address these methodological and practical challenges, researchers have proposed adopting a Latent Profile Analysis (LPA) approach. Compared with traditional variable-centered approaches, it offers a more realistic representation of complex phenomena [13]. Latent profile analysis (LPA) is approach that identifies subgroups of individuals based on their response patterns to continuous manifest indicators. Like latent class analysis (LCA), LPA emphasizes heterogeneity among individuals, but it is specifically suited for continuous data, whereas LCA is used for categorical data [14]. By leveraging objective statistical criteria, LPA quantifies individual differences more precisely and captures multidimensional qualitative distinctions, maximizing inter-group heterogeneity and intra-group homogeneity. This method offers significant advantages for identifying psychological and behavioral problems [5]. Using latent profile analysis (LPA), this study examines individual heterogeneity to identify distinct subgroups of suicide risk among college students, offering a practical tools for university mental health interventions.

Based on the stress-diathesis model and latent profile analysis (LPA) methodology, this study proposes the following hypothese: college students at risk for suicide constitute a heterogeneous population wherein distinct subgroups (latent profiles) will emerge based on differential response patterns to key continuous indicators derived from the Stress-Diathesis Model—specifically stress factors, diathesis factors, and protective factors. The research objectives are twofold: First, to determine whether these latent profiles exhibit statistically significant differences in the severity of self-reported suicidal ideation and behavior, thereby enabling precise stratification of suicide risk levels; second, to elucidate the distinct configurations of stress, diathesis, and protective factors characterizing each profile, with the ultimate aim of informing the development of targeted intervention protocols and management strategies.

## 2. Methods

### 2.1. Participants

All first-year students are required to use the “Xin Hai Navigation Suicide Risk Assessment and Crisis Intervention System” [15] to screen for suicide-related risk factors and evaluate their potential suicide risk levels. The more suicide-related risk factors an individual objectively has and the more severe these factors are, the higher the potential suicide risk may be. The measurement tool consists of five parts, comprising a total of 88 items, including the Personal Growth Experience Questionnaire, Neuroticism Personality Trait Test, Adolescent Life Events Scale, Social Support Rating Scale, and Beck Depression Inventory. The results are divided into five levels: (1) Total score ≥7, extremely high potential suicide risk; (2) Total score ≥5, relatively high potential suicide risk; (3) Total score ≥3, moderate potential suicide risk; (4) Total score ≥1, relatively low potential suicide risk; (5) Total score <1, very low potential suicide risk (normal). (The total score is derived from the weighted scores of each factor, with a range of 0–9 points.) Based on psychological census data from a vocational college, this study analyzed subjects with extremely high or relatively high potential suicide risks(Total score ≥5), as well as those with prior suicidal experiences. The initial sample comprised 1,654 participants. After excluding 33 subjects due to high concealment scores and one due to missing data, the final sample included 1,620 participants (76.06% male, 23.94% female), aged between 16 and 23 years. The psychological census was administered during the first month of the academic year, organized by the school and conducted via networked computers under the supervision of counselors and mental health center staff. All participants provided informed consent, signed a consent form, and were briefed on the study’s purpose and significance. Students identified as high-risk received follow-up interventions from counselors and mental health professionals.The recruitment period of the participants for this study was from October to November 2024. This study received ethical approval from the Academic Ethics of Sichuan College of Architectural Technology (Approval ID: 20241001).

### 2.2. Procedure

The study employed cluster sampling to recruit 5,489 freshmen (males: 4,240) and 6,043 sophomores (males: 4,705) from a higher vocational college for computerized group administration. Supervised by rigorously trained postgraduate psychology students, participants received standardized instructions emphasizing truthful responses without right/wrong answers. After confirming comprehension, participants proceeded to designated websites for sequential item completion without backtracking or omissions. The untimed assessment required approximately 40 minutes to complete fully. System-assessed participants designated as very high-risk or high-risk for suicide (n = 1,620) subsequently underwent latent profile analysis.

### 2.3. Research tools

#### 2.3.1. Measurement of stress factors.

The Adolescent Life Events Scale (ASLEC) was employed to assess the stressful life events that college students have recently encountered and to evaluate the frequency and intensity of these negative life events. The Adolescent Life Events Scale (ASLEC), developed by Liu et al. (1997), is a self-report questionnaire comprising 27 negative life events that may elicit psychological and physiological responses in adolescents [14]. The scale includes six factors: interpersonal relationships, academic pressure, punishment, loss of relatives or property, health and adaptation problems, and other stressors, which collectively capture common psychosocial stressors among adolescents. The assessment period can be set at 3, 6, 9, or 12 months, depending on research objectives. In this study, a 6-month evaluation period was adopted. Participants first indicated whether each event occurred within the specified timeframe. If an event occurred, its psychological impact was rated on a 5-point scale: no impact (1), mild (2), moderate (3), severe (4), or extremely severe (5). Statistical measures include event frequency and stimulus intensity, with non-occurring events classified as “no impact.” The total stimulus intensity is calculated as the cumulative score across all events. The ASLEC demonstrates strong reliability and validity, with a Cronbach’s alpha of 0.849 and a split-half reliability coefficient of 0.881, indicating excellent internal consistency.

#### 2.3.2. Symptom factors.

In this study, the Symptom Checklist-90 (SCL-90), developed by Derogatis, L. R. in 1977, was used to assess participants’ psychological health [16]. The SCL-90 is a widely utilized self-report measure designed to evaluate the severity of psychological symptoms experienced over the past week. The scale comprises 90 items across nine primary dimensions: obsessive-compulsive symptoms, anxiety, depression, hostility, phobic anxiety, paranoid ideation, psychoticism, somatization, and interpersonal sensitivity. The Chinese version of the SCL-90 demonstrates good reliability, with an internal consistency coefficient of 0.83 [16].

The current depression levels of college students were assessed using the Chinese version of the Beck Depression Inventory-II (BDI-II). Originally developed by Aaron T. Beck in 1961, the BDI-II is a self-report measure designed to evaluate the severity of depressive symptoms. In 1996, Beck and colleagues revised the original BDI to align with the diagnostic criteria for depression outlined in the fourth edition of the Diagnostic and Statistical Manual of Mental Disorders (DSM-IV) [17]. The revised BDI-II comprises 21 items assessing emotional, cognitive, and physiological symptoms and is widely utilized in both clinical and research contexts for individuals aged 13–80. The Chinese version of the BDI-II demonstrates strong psychometric properties, with a Cronbach’s α coefficient of 0.85, indicating excellent internal consistency [18]. Participants rated each item on a 4-point scale (0–3) based on their experiences over the past two weeks, and the total score was calculated as the sum of these item scores.

#### 2.3.3. Susceptibility factors.

The Personal Growth Experience Questionnaire is designed to comprehensively assess various aspects that may influence an individual’s development. It delves into the domains of family function, family economic status, parental parenting styles, childhood experiences, substance abuse, personal history of mental illness, family suicide history, and personal experience of attempted suicide. Comprising a total of 19 items, this questionnaire originates from the “Heart Sea Crisis Intervention System” and serves as a valuable tool for understanding the intricate interplay of factors that shape an individual’s growth trajectory. The scoring rules of the scale are as follows: The scale consists of multiple-choice questions, each providing several options, typically “yes” or “no,” with some questions offering more options (e.g., “good/average/poor/unable to answer”). Scoring is assigned based on the selected options. For instance, “yes” is scored as 1 point, and “no” is scored as 0 points. For questions with multiple options, such as “good/average/poor/unable to answer,” the scores are 0, 1, 2, and 3 points, respectively. The total score is calculated by summing the points of all questions, which is then used to assess the individual’s level of potential risk.

The Neuroticism (N) dimension from the revised Eysenck Personality Questionnaire-Revised Short Scale in the Chinese language (EPQ-RSC) was employed to evaluate the emotional stability of college students. The EPQ-RSC, originally developed by renowned British psychologists Hans J. Eysenck and Sybil B. G. Eysenck, is grounded in the well-established three-dimensional personality theory [19]. The N dimension specifically measures individual differences in emotional stability versus instability through a series of carefully constructed declarative items, assessing tendencies toward anxiety, moodiness, irritability, and emotional reactivity. The Chinese adaptation of the EPQ-RSC has demonstrated robust psychometric properties in previous validation studies. Specifically, the N subscale has shown satisfactory reliability coefficients (Cronbach’s α > 0.85) and strong construct validity in Chinese populations [20].

#### 2.3.4. Protective factors.

The assessment of protective factors in this study incorporated the Social Support Rating Scale (SSRS), a psychometrically validated instrument originally developed by Xiao (1986) to measure individuals’ social support systems [21]. This 10-item scale comprises three theoretically grounded and empirically validated dimensions: (1) objective support (3 items), which quantifies tangible assistance and network resources; (2) subjective support (4 items), which assesses perceived availability and adequacy of support; and (3) support utilization (3 items), which evaluates individuals’ propensity to actively seek and utilize available support resources. The SSRS has demonstrated robust psychometric properties in previous validation studies, with internal consistency coefficients (Cronbach’s α) ranging from 0.82 to 0.89 across different populations.

#### 2.3.5 Dependent variable: Adolescent Suicidal Tendency Scale (ASTS).

The Adolescent suicidal tendency Scale (ASTS), developed by Si et al. (2014), was employed to assess the current level of suicidal tendency among college students [22]. The ASTS evaluates suicidal tendency through three dimensions: despair, suicide identity, and suicide preparation. The scale demonstrates strong psychometric properties, with a Cronbach’s alpha coefficient of 0.89, indicating high internal consistency. Previous studies have confirmed its reliability and validity in college student populations, supporting its utility as a robust tool for measuring suicidal tendency in this demographic.

### 2.4. Statistical methods for data analysis

The data analysis was conducted in three main stages. First, correlation analysis was performed to examine the relationships among the measurement indicators. Second, regression analysis was employed to investigate the influence of various factors on suicidal tendencies. Specifically, uni-variate regression models were established for each psychological measurement indicator to assess their individual predictive power on suicidal tendencies. Given the non-normal distribution of certain variables (e.g., stress, growth experiences), the significance of all regression coefficients was tested using the Bootstrap method with 1,000 resamples. When dealing with non-normal data and small sample sizes, traditional methods may not provide accurate estimates of standard errors or confidence intervals. Bootstrapping can be particularly useful in these cases because it does not rely on the assumption of normality [5]. Latent profile analysis (LPA) was employed to identify distinct risk subtypes in college students’ suicide risk, based on the method’s capacity to model unobserved population heterogeneity using categorical latent variables. The model fitting process began with a null model (one latent class) and incrementally increased the number of latent classes. Model parameters were estimated, and adaptability tests were performed to identify the optimal model. LPA estimates the probability of individuals belonging to specific risk groups, with classification accuracy evaluated using statistical indices such as log-likelihood (LL), Akaike Information Criterion (AIC), Bayesian Information Criterion (BIC), Lo-Mendell-Rubin Likelihood Ratio Test (LMRT), and Entropy. A model with higher Entropy, lower AIC and BIC values, and significant LMRT results indicates better fit. Entropy, ranging from 0 to 1, reflects classification accuracy; values above 0.8 indicate over 90% accuracy, while values of 0.6 suggest approximately 20% misclassification [23]. Data analysis was performed using R 4.4.2 and SPSS 21 software.

## 3. Results

### 3.1. Correlation analysis of measurement indicators

Appendix 1 presents the correlation analysis results for the 19 measurement indicators examined in this study. The findings indicate that suicidal tendency exhibits positive correlations with all measurement indicators except social support, which shows a negative correlation. Stress factors (life events) demonstrate positive correlations with all indicators except growth experience. Symptom factors (e.g., somatization, obsessive-compulsive symptoms, anxiety, depression) display positive correlations with all indicators except social support, which again shows a negative correlation. Growth experience is positively correlated with all indicators except neuroticism and social support. Neuroticism exhibit positive correlations with all indicators except growth experience and social support. Social support shows a positive correlation with life events but negative correlations with hopelessness, suicidal ideation, suicidal preparation, suicidal tendency, obsessive-compulsive symptoms, interpersonal sensitivity, depression, anxiety, hostility, phobia, paranoia, psychosis, diet, sleep problems, and the Beck Depression Index. No significant correlations were found between social support and somatization, growth experience, or neuroticism.

### 3.2. Predictive variables of suicidal tendency

#### 3.2.1. Regression analysis of stress factors on suicidal tendency.

A univariate regression analysis was conducted with life events as the independent variable and suicidal tendency as the dependent variable. The results, presented in Table 1, indicate that life events significantly predict suicidal tendency (p < 0.01). The R² value (0.04) in our univariate regression analysis reflects inherent complexity of suicidal behavior as emphasized in our stress-diathesis framework, suicidal tendencies emerge from dynamic interactions between multiple risk and protective factors. Although the explanatory power of stress factors (R² = 0.04) for suicidal tendencies is statistically significant, it suggests the need to integrate other predictors (such as genetic markers) to construct a comprehensive prediction model.

**Table 1:** Regression analysis of stress factors on suicidal tendency.

	β	t	SE	R^2^
Negative life events	0.19^**^	7.66	0.03	0.04

#### 3.2.2. Regression analysis of symptom factors on suicidal tendency.

Univariate regression analyses were conducted using the symptom indicators from the SCL-90 and the Beck Depression Index as independent variables, with suicidal tendency as the dependent variable. The results, presented in Table 2, demonstrate that all symptom indicators significantly predict suicidal tendency (p < 0.01). Each factor was analyzed using univariate regression.

**Table 2:** Regression analysis of symptom factors on suicidal tendency.

	β	t	95% CI	R^2^
Somatization	0.44^**^	19.89	0.39, 0.49	0.2
Obsessive-compulsive symptoms	0.35^**^	14.82	0.30, 0.40	0.12
Interpersonal sensitivity	0.36^**^	15.56	0.31, 0.42	0.13
SCL-90 depression index	0.52^**^	24.6	0.48, 0.57	0.13
Anxiety	0.45^**^	20.08	0.40, 0.50	0.2
Hostility	0.44^**^	19.96	0.40, 0.49	0.2
Terror	0.41^**^	17.91	0.36, 0.46	0.17
Paranoia	0.40^**^	17.3	0.39, 0.46	0.16
Psychoticism	0.46^**^	20.77	0.34, 0.44	0.21
Diet, sleep, etc.	0.47^**^	21.13	0.41, 0.51	0.22
Beck depression index	0.43^**^	19.2	0.38, 0.49	0.19

#### 3.2.3. Regression analysis of susceptibility factors on suicidal tendency.

Univariate regression analyses were performed with growth experiences and neuroticism as independent variables and suicidal tendency as the dependent variable. The results revealed that both growth experiences and neuroticism significantly predicted suicidal tendency (p < 0.015).(See Table 3)

**Table 3:** Regression analysis of susceptibility factors on suicidal tendency.

	β	t	SE	R^2^
Growth experience	0.29**	12.39	0.02	0.09
Neuroticism	0.28**	11.78	0.02	0.08

#### 3.2.4. Regression analysis of protective factors on suicidal tendency.

A univariate regression analysis was conducted with social support as the independent variable and suicidal tendency as the dependent variable. The results demonstrated that social support significantly predicted suicidal tendency (p < 0.01), indicating an inverse relationship between the two variables. Specifically, higher levels of social support were associated with lower levels of suicidal tendency, highlighting the protective role of social support in mitigating suicide risk. (See Table 4)

**Table 4:** Regression analysis of protective factors on suicidal tendency.

	β	t	SE	R^2^
Social support	−0.19**	−7.77	0.02	0.04

Based on the aforementioned findings, this study identified 15 indicators associated with the suicide risk among college students, which were categorized into four dimensions: stress factor (1 indicator), symptom factors (11 indicators), susceptibility factors (2 indicators), and protective factors (1 indicator). Additionally, four indicators of suicidal tendency were included: despair, suicidal preparation, suicidal self-concept, and suicidal tendency. In total, 19 measurement indicators were selected to construct a latent profile model for the early warning of suicide risk. This integrative approach aims to provide a comprehensive framework for identifying and assessing the multifaceted nature of suicide risk within this population.

### 3.3. Latent profile model for college students’ suicide risk warning

The study examined latent profile models with 1–8 classes, and the fit indices are detailed in Table 5. Following recommendations for large samples (N > 1,000; Qiu, 2008), the Bayesian Information Criterion (BIC) served as the primary selection criterion. As shown in Table 6, while BIC values decreased monotonically from 87,615 (1-class) to 63,328 (8-class), the 3-class solution achieved the lowest classification error (0.048) among all models, significantly lower than adjacent solutions (2-class: 0.057; 4-class: 0.059). Concurrently, it yielded the highest entropy (0.95), indicating superior classification accuracy. The interpretability of the 3-profile structure further validated this selection: distinct risk strata (High, Moderate, and Low Risk) aligned with differential intervention needs, whereas the 4-class solution fragmented clinically coherent groups (e.g., splitting moderate-risk into non-actionable subtypes). Thus, the 3-class model optimally balanced statistical precision (BIC > 1,000) and theoretical utility, compared to the 2-class model.

**Table 5:** Comparison of model fit indices for latent profile analysis.

	classes	LL	BIC	AIC	Npar	Class.Error	Entropy
1	1-Cluster	−43667	87615	87410	87372	0	1
2	2-Cluster	−37571	75710	75295	75218	0.057055	0.94
3	3-Cluster	−34951	70758	70133	70017	0.04762136	0.95
4	4-Cluster	−33584	68313	67478	67323	0.05908392	0.94
5	5-Cluster	−32935	67303	66257	66063	0.07522486	0.93
6	6-Cluster	−32243	66207	64951	64718	0.06112645	0.93
7	7-Cluster	−32141	66292	64826	64554	0.07641217	0.92
8	8-Cluster	−31509	65316	63639	63328	0.06994656	0.9

**Table 6:** Average classification probabilities for latent profiles (Rows) by Participant Groups (Columns).

	C-1(%)	C-2(%)	C-3(%)
C1	97.89	2.11	0.00
C2	0.63	96.62	2.75
C3	0.00	3.42	96.58

The probability matrix for assignment to latent profiles (C1, C2, C3) and the corresponding latent classifications (C-1, C-2, C-3) is presented in Table 6. The average probability of classification accuracy for each latent category ranged from 96.58% to 97.89%, indicating high reliability in the assignment of college students to the three latent suicide risk profiles. These results demonstrate the robustness and precision of the 3-class latent profile model in classifying suicide risk among the studied population.

**Fig 1:**
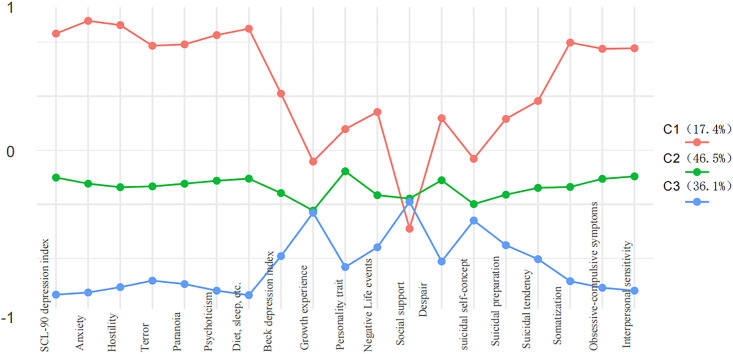
Latent profiles of suicide risk among participants.

Fig 1 visually presents the conditional probabilities of latent categories for predicting college students’ suicide risk in a line graph format, providing an intuitive representation of the patterns across categories. Higher conditional probabilities indicate stronger tendencies of participants in the corresponding latent category on the measured indicators, with the nature of each latent category defined by its high-probability features. As shown in Fig 1, the latent profile analysis identified three distinct suicide risk groups among college students. Group C1 (17.4%, high-risk) demonstrated significantly elevated scores relative to other groups across all psychological indicators, particularly showing the highest levels of neuroticism, depression, anxiety, eating disorders, sleep problems, interpersonal sensitivity, obsessive-compulsive symptoms, and somatization. Additionally, they showed higher scores in suicidal tendency, suicide identification, and suicide preparation, coupled with the lowest social support. Group C2 (46.5%, moderate-risk) exhibited intermediate scores, with neuroticism scores significantly lower than C1 but higher than C3, moderate symptom levels, and elevated suicidal tendencies. Group C3 (36.1%, low-risk) displayed the most adaptive pattern: highest social support, below-average scores on all symptoms, and the lowest suicidal tendencies/preparation. Crucially, each profile is defined by its multidimensional pattern across all indicators rather than any single measure. Neuroticism scores shows a decreasing gradient (C1 > C2 > C3), and social support shows the inverse pattern (C3 > C2 > C1), confirming robust inter-group distinctions.

## 4. Discussion

This study aimed to scientifically classify and manage assessment data following preliminary suicide risk assessment. The investigation primarily focused on the relationship between suicidal tendency and various psycho-social factors. The results demonstrated that suicidal tendency exhibited positive correlations with all measured factors except social support, aligning with previous research findings [5]. Specifically, the positive association between suicidal tendency and stress factors (negative life events) supports both the stress-diathesis model and existing empirical evidence [24]. Negative life events, including unemployment, family conflicts, and economic pressures, significantly elevate individuals’ suicidal self-concept and behavior by inducing feelings of helplessness and hopelessness, thereby exacerbating suicide risk [25]. Pleyard et al.’s cumulative situational risk model further elucidates this relationship, positing that negative life events accumulate or compound to increase risk, meaning that greater exposure to adverse situations leads to more severe individual impacts [26]. The positive correlation between symptomatic factors (anxiety, depression) and suicidal tendencies also aligns with the stress-diathesis model’s predictions. While suicide typically results from multiple factors, research indicates that up to 90% of completed suicides meet the criteria for mental disorders [27]. Consequently, addressing college students’ mental health, providing timely assistance for psychological distress, and alleviating their suffering constitute crucial components of suicide intervention strategies. Furthermore, the positive association between personality traits (neuroticism) and suicidal tendencies reinforces the diathesis-stress model’s premise regarding the significant role of intrinsic susceptibility traits in suicidal behaviors. Individuals with high neuroticism often experience emotion regulation difficulties, struggling to effectively manage and alleviate negative emotions. This predisposition makes them more susceptible to persistent states of anxiety, depression, or anger, and the chronic accumulation of these negative emotions can escalate psychological stress, thereby increasing suicide risk. Patients with greater neuroticism also manifest greater levels of hopelessness and, in turn, more suicidal behavior [28]. Notably, social support demonstrated a negative correlation with suicidal tendencies, indicating its protective role in buffering the adverse effects of stress factors and symptomatic factors on suicide risk. This finding corroborates Hong’s (2022) research, underscoring the critical importance of social support in suicide prevention efforts [12].

Life events, as primary stress factors, demonstrated positive correlations with all measures except growth experiences. This indicates that negative life events not only directly elevate an individual’s psychological stress but may also indirectly increase suicide risk by exacerbating psychological symptoms (e.g., depression, anxiety) and reinforcing negative personality traits (e.g., neuroticism). The empirical evidence lends robust support to the core premise of the stress-diathesis model, which posits that the interaction between external stress factors and internal susceptibility traits constitutes a key mechanism underlying suicidal behavior. Symptom factors (e.g., somatization, obsessive-compulsive symptoms, anxiety, depression) exhibited positive correlations with all measures except social support, further validating the significant role of psychological symptoms in suicide risk. Notably, depression and anxiety showed particularly strong associations with suicidal tendencies, suggesting that these symptoms may serve as critical early warning indicators of suicidal behavior. Additionally, the positive correlation between symptom factors and neuroticism implies that psychological symptoms may further elevate suicide risk by reinforcing negative cognitive patterns and non-adaptive emotional responses. Growth experiences were positively correlated with other measures except neuroticism and social support, indicating that early life experiences may influence adult mental health by shaping psychological traits and coping mechanisms. However, the lack of significant correlation between formative experiences and neuroticism suggests that personality development is influenced by multiple factors rather than being solely determined by early experiences. The observed positive correlation between social support and stressful life events suggests a potential association wherein individuals reporting more life events also report higher social support, though the directionality and underlying mechanisms require further investigation. Conversely, the negative correlations between social support and hopelessness, suicidal self-concept, and suicide preparation underscore the protective role of social support in mitigating negative emotions and reducing suicide risk. These results emphasize the importance of strengthening social support networks as a critical component of suicide prevention and intervention strategies.

This study identifies and analyzes predictive factors of suicidal tendencies among college students. Life events, as primary stress factors, significantly predict suicidal tendencies, aligning with the stress-diathesis model. This suggests that negative life events, such as academic pressure, family conflicts, and interpersonal problems, may elevate suicide risk by intensifying psychological stress and negative emotions. These findings underscore the importance of prioritizing students who have experienced significant negative life events in university mental health initiatives, ensuring timely psychological support and intervention. The SCL-90 symptoms (e.g., somatization, obsessive-compulsive symptoms, anxiety, depression) and the Baker Depression Index emerged as significant predictors of suicidal intention, further affirming the central role of psychological symptoms in suicide risk. Notably, the strong predictive power of depression and anxiety symptoms highlights their potential as critical early warning indicators of suicidal behavior. Additionally, the predictive effects of obsessive-compulsive symptoms and interpersonal sensitivity suggest that diverse mental health issues may contribute to suicide risk through distinct mechanisms. Consequently, suicide prevention efforts should emphasize the early identification and intervention of psychological symptoms. Growth experiences significantly predict suicidal tendencies, indicating that early life experiences, such as childhood trauma and family environment, may shape individuals’ psychological traits and coping styles, thereby influencing mental health outcomes in adulthood. This finding supports developmental psychology theories regarding the long-term impact of early experiences on psychological development. Therefore, mental health education and intervention programs should consider individuals’ growth backgrounds, particularly targeting students with adverse early experiences and providing tailored psychological support. neuroticism also significantly predict suicidal tendencies, consistent with the diathesis-stress model. Individuals with higher neuroticism may exhibit greater vulnerability to negative life events, increasing their susceptibility to suicidal ideation. These results highlight the importance of incorporating personality trait assessments into suicide risk evaluations, particularly for individuals with high neuroticism, low self-esteem, or similar traits, who may require enhanced psychological support and intervention. Social support significantly predicts suicidal intention, with higher levels of social support associated with lower suicidal tendencies. This underscores the protective role of social support in suicide prevention, as it may mitigate the adverse effects of stress factors and psychological symptoms by providing emotional support, enhancing psychological resilience, and improving coping mechanisms. Therefore, university mental health initiatives should focus on building and strengthening students’ social support networks through strategies such as peer support programs and faculty engagement to elevate students’ access to social support.

In this study, the potential profile analysis (LPA) method was employed to compare fit indices across different latent profiles, including BIC, LL, and Entropy. The three-class model was ultimately selected as the optimal solution, consistent with Qiu Haozheng’s (2008) recommendations for model selection in large-sample scenarios [29]. The three latent profiles demonstrated clear classification, distinct characteristic indices for each category, and high classification probabilities (ranging from 96.58% to 97.89%), indicating strong reliability and discriminative validity of the model. High-risk group (C1, 17.4%): This group exhibited elevated scores on psychological symptoms such as depression, anxiety, interpersonal sensitivity, and obsessive-compulsive tendencies, alongside higher scores on suicidal intention, suicidal self-concept, and suicidal preparation, indicating a significantly elevated suicide risk. These findings align with prior research demonstrating a strong association between suicide risk and psychological symptoms. For instance, a meta-analysis by Franklin et al. (2017) identified depression, anxiety, and feelings of hopelessness as core predictors of suicide risk [30]. Moderate-risk group (C2, 46.5%): This group showed a higher propensity for neuroticism and feelings of hopelessness. While they also displayed an elevated propensity for suicide, their overall risk was lower than that of the high-risk group. The existence of this category suggests that neuroticism and psychological resilience may play a crucial moderating role in mitigating suicide risk. Low-risk group (C3, 36.1%): This group demonstrated a higher propensity for social support and suicide identification but scored lower on other psychological symptoms and suicidal tendencies, indicating a reduced risk of suicide. The protective role of social support is prominently reflected in this category, underscoring its importance in buffering against suicide risk.

The theoretical significance of this study lies in its construction of a suicide risk early warning model using multi-dimensional indicators, offering a novel perspective for the quantitative assessment of suicide risk.This study pioneers the application of LPA-derived risk phenotypes in suicide prevention, moving beyond variable-centered approaches to capture synergistic risk configurations. The findings also support the applicability of the Diathesis-Stress Model to suicide risk. Practically, by identifying high-risk groups, colleges and universities can implement more targeted mental health education and crisis interventions, optimize resource allocation, and enhance intervention efficacy.

This study provides a theoretical foundation for the early identification and intervention of suicide risk among college students. For instance, targeting individuals with high stress and elevated symptom levels can reduce suicide risk by strengthening social support and alleviating psychological symptoms. This study has several important limitations that warrant careful consideration. First, the correlational design inherently precludes causal inferences, necessitating future longitudinal studies to elucidate temporal dynamics and directional relationships among variables. Second, potential unmeasured genetic confounders cannot be ruled out, possibly contributing to spurious associations. Third, reliance on self-reported measures, despite implemented validity checks, introduces risks of common method variance and recall bias, while limitations in sample representativeness and measurement tools may constrain generalizability.

In conclusion, these exploratory findings suggest potential relationships between self-reported psychosocial factors and suicidal tendencies among college students. While the derived profiles require replication in independent samples and validation through multi-method assessments, the current results may inform preliminary considerations for university mental health screening. Suicide is a critical indicator of public mental health and a key measure of the effectiveness of public health strategies aimed at improving population mental health. Suicide prevention should encompass three levels: Universal Prevention: Aimed at the entire population, these strategies seek to reduce risk factors and enhance protective factors, regardless of individual suicide risk. The advantage of such approaches is their broad reach, addressing suicide risk at its root. Selective Prevention: Targeted at subgroups with elevated suicide risk based on biological, social, and environmental factors (e.g., unemployment, marital dissolution, low income, or sexual abuse). While not all members of these subgroups are at risk, the prevalence of potential suicides is higher, allowing for more effective, goal-directed interventions to mitigate specific risk factors. Targeted Prevention: Focused on individuals exhibiting signs of suicidal behavior or conditions associated with suicide. Strategies may include care management for individuals discharged from inpatient facilities, psychiatric treatment, and cognitive-behavioral skills groups [31]. Based on the three distinct risk profiles identified in this study, we propose the following potential intervention strategies for researchers and clinicians considering applying these profiles to guide treatment allocation: C1: Clinical crisis intervention (combining Cognitive Behavioral Therapy (CBT) and medication) is suggested for the high-risk group. This integrated approach is recommended due to the heightened severity and potential acuity of symptoms in this group, where CBT can address maladaptive cognitions and behaviors, while medication may be necessary for rapid symptom stabilization and management of underlying biological factors. C2: Group counseling focused on Mindfulness-Based Stress Reduction (MBSR) is proposed for the medium-risk group. MBSR is well-suited for this profile as it effectively enhances coping skills, emotional regulation, and resilience in individuals experiencing significant but less acute distress [31], aligning with the characteristics observed in this group. C3: Facilitating the establishment of peer support networks is recommended for the low-risk group. This lower-intensity intervention capitalizes on existing strengths and social resources within this group, fostering mutual support and potentially preventing escalation of risk [31], which is congruent with their profile characteristics.

## Supporting information

S1 Appendix Correlation Matrix of Measurement Indicators. (DOC)

S1 Data (XLSX)

S1 File STROBE flow diagram. (DOCX)
