# Supplementary material for: Suicide risk characteristics of vocational college students: A latent profile analysis
Source: PLoS One. 2025 Oct 31;20(10):e0333303. doi: 10.1371/journal.pone.0333303 (PMC12578167; doi:10.1371/journal.pone.0333303)
Supplement: S1 Appendix — (DOC) [file pone.0333303.s001.doc]

| Appendix 1 Correlation Matrix of Measurement Indicators | | | | | | | | | | | | | | | | | | | |
| --- | --- | --- | --- | --- | --- | --- | --- | --- | --- | --- | --- | --- | --- | --- | --- | --- | --- | --- | --- |
|  | 1 | 2 | 3 | 4 | 5 | 6 | 7 | 8 | 9 | 10 | 11 | 12 | 13 | 14 | 15 | 16 | 17 | 18 | 19 |
| 1. Life events |  |  |  |  |  |  |  |  |  |  |  |  |  |  |  |  |  |  |  |
| 2. Despair | 0.184** |  |  |  |  |  |  |  |  |  |  |  |  |  |  |  |  |  |  |
| 3. Suicidal identity | 0.053* | 0.115** |  |  |  |  |  |  |  |  |  |  |  |  |  |  |  |  |  |
| 4. Suicidal preparation | 0.149** | 0.355** | 0.351** |  |  |  |  |  |  |  |  |  |  |  |  |  |  |  |  |
| 5. Suicidal tendency | 0.187** | 0.688** | 0.558** | 0.873** |  |  |  |  |  |  |  |  |  |  |  |  |  |  |  |
| 6. Somatization | 0.399** | 0.328** | 0.160** | 0.412** | 0.443** |  |  |  |  |  |  |  |  |  |  |  |  |  |  |
| 7. Obsessive-compulsive symptoms | 0.369** | 0.421** | 0.021 | 0.253** | 0.346** | 0.657** |  |  |  |  |  |  |  |  |  |  |  |  |  |
| 8. Interpersonal sensitivity | 0.389** | 0.455** | 0.042 | 0.244** | 0.361** | 0.606** | 0.789** |  |  |  |  |  |  |  |  |  |  |  |  |
| 9. SCL-90 depression index | 0.398** | 0.563** | 0.161** | 0.372** | 0.522** | 0.714** | 0.814** | 0.810** |  |  |  |  |  |  |  |  |  |  |  |
| 10. Anxiety | 0.441** | 0.425** | 0.136** | 0.360** | 0.447** | 0.813** | 0.752** | 0.754** | 0.824** |  |  |  |  |  |  |  |  |  |  |
| 11. Hostility | 0.423** | 0.402** | 0.145** | 0.369** | 0.444** | 0.769** | 0.699** | 0.710** | 0.773** | 0.888** |  |  |  |  |  |  |  |  |  |
| 12. Terror | 0.363** | 0.339** | 0.152** | 0.348** | 0.407** | 0.673** | 0.648** | 0.673** | 0.697** | 0.749** | 0.701** |  |  |  |  |  |  |  |  |
| 13. Paranoia | 0.370** | 0.386** | 0.102** | 0.320** | 0.395** | 0.667** | 0.682** | 0.733** | 0.741** | 0.766** | 0.736** | 0.616** |  |  |  |  |  |  |  |
| 14. Psychoticism | 0.418** | 0.436** | 0.119** | 0.380** | 0.459** | 0.707** | 0.767** | 0.762** | 0.787** | 0.792** | 0.752** | 0.709** | 0.681** |  |  |  |  |  |  |
| 15. Diet, sleep, etc. | 0.425** | 0.446** | 0.122** | 0.382** | 0.465** | 0.757** | 0.821** | 0.816** | 0.841** | 0.835** | 0.797** | 0.762** | 0.717** | 0.958** |  |  |  |  |  |
| 16. Beck depression index | 0.333** | 0.404** | 0.234** | 0.303** | 0.431** | 0.449** | 0.419** | 0.432** | 0.570** | 0.504** | 0.486** | 0.429** | 0.432** | 0.471** | 0.483** |  |  |  |  |
| 17. Growth experience | 0.02 | 0.074** | 0.176** | 0.342** | 0.294** | 0.196** | 0.127** | 0.094** | 0.137** | 0.146** | 0.146** | 0.151** | 0.125** | 0.158** | 0.159** | 0.067** |  |  |  |
| 18. Personality trait | 0.224** | 0.317** | 0.089** | 0.190** | 0.281** | 0.369** | 0.420** | 0.437** | 0.461** | 0.461** | 0.456** | 0.397** | 0.390** | 0.394** | 0.419** | 0.300** | 0.042 |  |  |
| 19. Social support | 0.144** | -0.176** | -0.137** | -0.119** | -0.189** | -0.033 | -0.056* | -0.084** | -0.130** | -0.052* | -0.052* | -0.074** | -0.091** | -0.117** | -0.114** | -0.150** | -0.036 | -0.013 |  |
| Note. *p < .05, **p < .01; n = 1,620. The same applies to subsequent tables. | | | | | | | | | | | | | | | | | | | |
