# Supplementary material for: Suicide risk characteristics of vocational college students: A latent profile analysis
Source: PLoS One. 2025 Oct 31;20(10):e0333303. doi: 10.1371/journal.pone.0333303 (PMC12578167; doi:10.1371/journal.pone.0333303)
Supplement: S1 File — (DOCX) [file pone.0333303.s003.docx]

Vocational college students: N = 11,532 (77.57% male)

Complete the suicide risk screening test in the “Xin Hai Navigation Suicide Risk Assessment and Crisis Intervention System.”

9912 participants (77.13% male)

Participants with a total score of less than 5 were excluded.

(This indicates a potential for suicide risk that is moderate, or even very low.)

1,620 participants (76.06% male)

Participants with a total score of 5 or above were included.

(This indicates a high potential for suicide risk.)

A Latent Profile Analysis
